# Differential plague susceptibility in species and populations of prairie dogs

**DOI:** 10.1002/ece3.5684

**Published:** 2019-10-02

**Authors:** Robin E. Russell, Daniel W. Tripp, Tonie E. Rocke

**Affiliations:** ^1^ National Wildlife Health Center U.S. Geological Survey Madison WI USA; ^2^ Wildlife Health Program Colorado Parks and Wildlife Fort Collins CO USA

**Keywords:** disease susceptibility, plague, prairie dogs, survival analyses

## Abstract

Laboratory trials conducted over the past decade at U.S. Geological Survey National Wildlife Health Center indicate that wild populations of prairie dogs (*Cynomys* spp.) display different degrees of susceptibility to experimental challenge with fully virulent *Yersinia pestis*, the causative agent of plague. We evaluated patterns in prairie dog susceptibility to plague to determine whether the historical occurrence of plague at location of capture was related to survival times of prairie dogs challenged with *Y. pestis*. We found that black‐tailed prairie dogs (*Cynomys ludovicianus*) from South Dakota (captured prior to the detection of plague in the state), Gunnison's prairie dogs (*Cynomys gunnisoni*) from Colorado, and Utah prairie dogs (*Cynomys parvidens*) from Utah were most susceptible to plague. Though the susceptibility of black‐tailed prairie dogs in South Dakota compared with western locations supports our hypothesis regarding historical exposure, both Colorado and Utah prairie dogs have a long history of exposure to plague. It is possible that for these populations, genetic isolation/bottle necks have made them more susceptible to plague outbreaks.

## Graphical abstract

We conduct meta‐analyses of the responses of prairie dogs (*Cynomys* spp.) from different populations across the western United States to plague challenge. We hypothesized that historical exposure to plague would affect the susceptibility of prairie dog populations to plague. We conclude that though historical exposure may play a role that connectivity of populations is also likely to affect a population's response to plague.

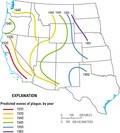

## 1 INTRODUCTION

**Figure 1:**
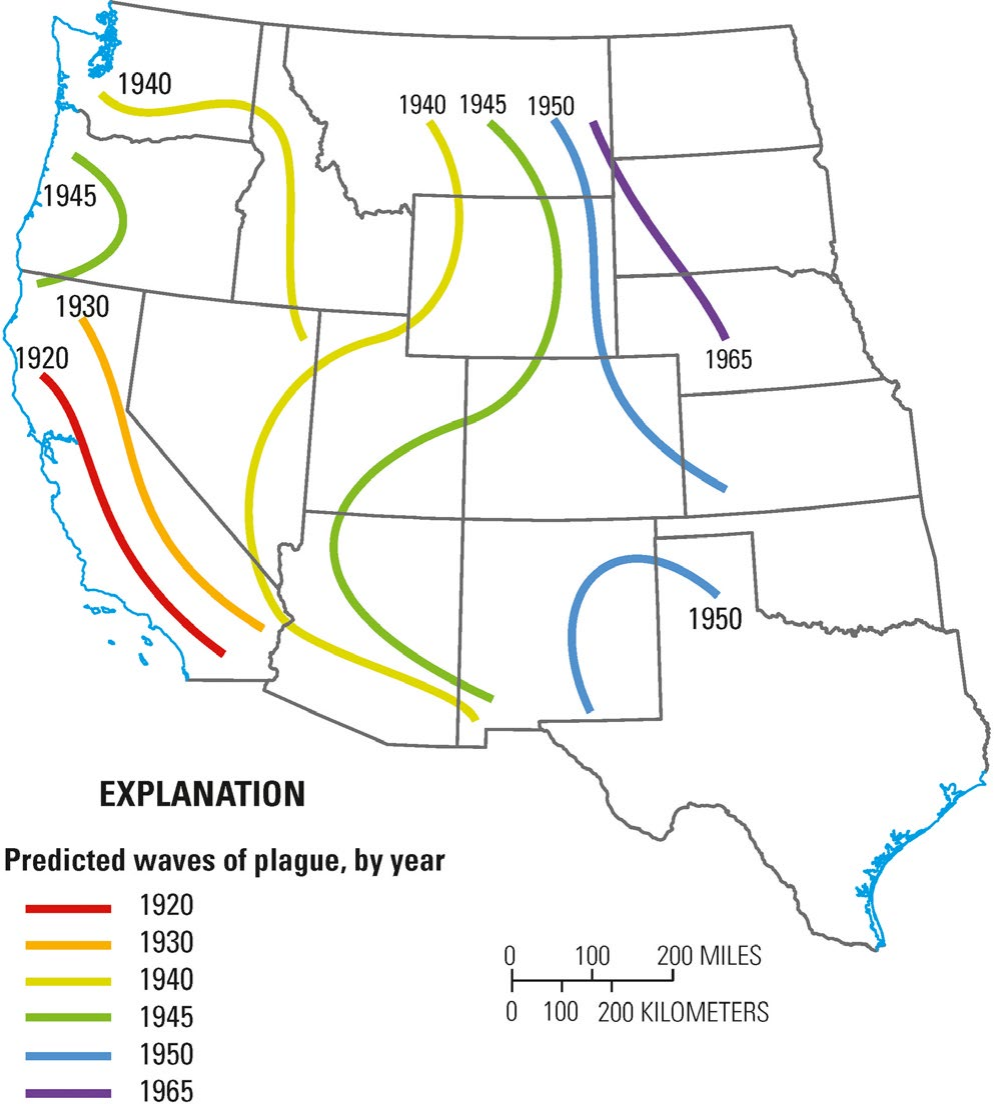
Map of the western United States showing historical plague spread indicating the decade when plague was first detected in a state, based on Adjemian et al., 2007, used with permission (Abbott & Rocke, 2012)

Plague, caused by the bacterium *Yersinia pestis*, periodically causes disease outbreaks in the western United States, resulting in widespread mortality of many native rodent species. Prairie dogs (*Cynomys* spp.) and ground squirrels (*Spermophilus* spp.) are highly susceptible to plague, with outbreaks often causing >90% mortality in affected colonies. Some studies have suggested that plague may also occur in prairie dog colonies at enzootic levels (Matchett, Biggins, Carlson, Powell, & Rocke, 2010) and/or that plague may persist in prairie ecosystems at low levels for long periods of time prior to an outbreak occurring (St. Romain, Tripp, Salkeld, & Antolin, 2013). Thus, the effects of plague may be more widespread and longer term than observations of outbreaks would imply. Plague was likely introduced into wild rodent populations in the United States by rats (*Rattus rattus*) arriving on ships from China, where the last worldwide pandemic of plague originated at the end of the 19th century (Echenberg, 2002). Historical literature indicates that plague was first confirmed in wild rodent populations in counties surrounding San Francisco Bay, California, in 1908 (Eskey & Haas, 1940). From there, plague moved eastward to the 101st meridian by the 1940s–1950s (Adjemian, Foley, Gage, & Foley, 2007; Barnes, & Poland 1983; Figure 1). More recently, plague has spread to the southwestern portion of South Dakota, where the disease was first recorded in 2004 (Figure 1).

Although mass mortality is common in prairie dogs during plague outbreaks, lowered susceptibility to plague has been observed in some populations of prairie dogs. For example, Rocke et al. (2012) noted differential survival rates in experimentally challenged black‐tailed prairie dogs (*Cynomys ludovicianus*), with animals from Texas and Colorado surviving at higher rates compared with animals from South Dakota where plague had not occurred previously. Pauli, Buskirk, Williams, and Edwards (2006) also noted that black‐tailed prairie dogs were capable of surviving plague outbreaks and regrouping into new coteries. This conclusion was inferred from genetic markers in a separate postplague population from Boulder County, Colorado (Sackett et al., 2014).

Differential plague susceptibility has been observed in other rodent species as well, including northern grasshopper mouse (*Onychomys leucogaster*; Thomas et al., 1998), California vole (*Microtus californicus*; Hubbert & Goldenberg, 1970), rock squirrel (*Spermophilus variegatus*; Quan, Barnes, Carter, & Tsuchiya, 1985), and wild populations of black rat (*R. rattus*; Tollenaere et al., 2010). Populations of northern grasshopper mouse in the United States (Thomas et al., 1998) and black rat in Madagascar (Tollenaere et al., 2010) displayed differential susceptibility based on the location of each population. Populations of northern grasshopper mouse from the Pawnee National Grassland in Colorado had lower mortality rates when challenged with plague versus populations from central Oklahoma (Thomas et al., 1998) where plague has not been recorded. Likewise, plague susceptibility differed between black rat populations sampled in the central highlands (where *Y. pestis* is present) versus a low altitude plague‐free area (Tollenaere et al., 2010). Both studies concluded that historical exposure to plague outbreaks was likely responsible for the differences in observed survival rates.

Other factors potentially influencing susceptibility to disease in wildlife populations include host genetic structure (Altizer, Harvell, & Friedle, 2003; Jousimo et al., 2014; O'Brien & Evermann, 1998). Though Hess (1996) demonstrated that highly connected populations are more likely to succumb to an epidemic due to host and pathogen movement through a population, other studies have suggested that connected populations may be more genetically diverse and display higher levels of pathogen resistance (Jousimo et al., 2014). Populations that have low genetic diversity or have experienced a genetic bottleneck may be less resistant to disease or conversely, if the bottleneck was caused by an epidemic, survivors may be more resistant to subsequent outbreaks (O'Brien & Evermann, 1998). Spielman, Brook, Briscoe, and Frankham (2004) demonstrated that the loss of specific “disease resistance” genes, rather than inbreeding itself was responsible for increased disease susceptibility in inbred fruit flies (*Drosophila melanogaster*). For prairie dogs, elevation may provide a barrier that influences the level of connectedness in populations (Sackett et al., 2014). Prairie dogs occur across a wide range of elevations, and it is possible that higher elevation populations are more genetically isolated and therefore less resistant to disease.

Currently, there is debate regarding the transmission dynamics of plague between prairie dog colonies. Previous studies have indicated a role for small mammals, mesocarnivores (Barnes, 1982; Salkeld, Salathe, Stapp, & Jones, 2010; Salkeld & Stapp, 2006), or prairie dogs (Cully & Williams, 2001; Savage, Reich, Hartley, Stapp, & Antolin, 2011) in transporting plague between disparate colonies. If some prairie dogs are less susceptible to plague, (i.e., it takes a larger dose of plague to cause mortality), it is possible that prairie dogs themselves are acting either as maintenance hosts or transporters of plague in prairie ecosystems (George, Webb, Pepin, Savage, & Antolin, 2013).

*Yersinia pestis* challenge studies have been ongoing at the U.S. Geological Survey National Wildlife Health Center (USGS NWHC) to support the development of a bait‐delivered sylvatic plague vaccine (SPV) for prairie dogs (e.g., Rocke et al., 2010; Rocke, Smith, Stinchcomb, & Osorio, 2008; Rocke et al., 2012). We consolidate data from control animals (plague inoculated but not vaccinated) across studies to evaluate relative plague susceptibility between study populations and to determine whether our data provide support for the idea that prairie dogs can develop some level of resistance to plague. We then explore potential factors associated with observed plague susceptibility to provide hypotheses for future studies.

## 2 METHODS

We consolidated data from several published and unpublished studies (Table 1), in which prairie dogs were challenged with virulent *Y. pestis* via subcutaneous injection of 3,500 mouse 50% lethal doses (MLD50) to assess the ability of SPV to protect prairie dogs (see specific publications listed in Table 1 for details, Rocke & Russell, 2019). All experiments took place at the USGS NWHC under the supervision of Dr. Rocke, using *Y. pestis* challenge inoculum derived from a single isolate (CO92) and with the same personnel responsible for inoculum preparation and injections of *Y. pestis*. These data included a total of 305 prairie dogs that were challenged but not vaccinated (positive controls) or received placebo vaccine baits. One hundred Gunnison's prairie dogs (*Cynomys gunnisoni*; 47 juvenile, 53 adult), from two states, and 143 adult black‐tailed prairie dogs from three states, were included from previous publications, as well as data from two unpublished studies, including 21 adult Utah prairie dogs (*Cynomys parvidens*) and 15 adult white‐tailed prairie dogs (*Cynomys leucurus*) from Colorado (Table 1). In addition, we included one group of 10 adult white‐tailed prairie dogs from the same Colorado population that were challenged with 10× the standard dose (35,000 MLD50). Previous research indicated no significant differences between survival rates of unvaccinated plague‐challenged juvenile or adult prairie dogs; therefore, we combine all prairie dogs for the purposes or our analyses.

**Table 1:** List of published data used in this study, including the state where the individual prairie dogs were collected, the number of animals challenged with *Yersina pestis* (*N*), year of capture, and elevation in meters (m) of capture location

Reference source	Location	Species	*N*	Year of capture	Elevation (m)
Mencher et al. (2004)	South Dakota	BT	18	2001	<1,500
Rocke et al. (2008)	South Dakota	BT	19	2004	871
Rocke et al. (2010)	South Dakota	BT	17	2007	1,035
Rocke et al. (2012)	Colorado	BT	24	2003	1,597
Rocke et al. (2012)	South Dakota	BT	22	2003	947
Rocke et al. (2012)	Texas	BT	15	2003	942
Rocke, Kingstad‐Bakke, Berlier, and Osorio (2014)	South Dakota	BT	28	2010	1,035
Busch et al. (2013)	Arizona	GU	60	2009	1,584 and 1,710
Rocke et al. (2015)	Colorado	GU	40	2011	2,375 and 2,040
Rocke unpublished	Utah	UT	21	2010	>2,000
Rocke unpublished	Colorado	WT	15	2011	1,875
Rocke unpublished^a^	Colorado	WT	10	2011	187

Abbreviations: BT, black‐tailed prairie dog (*Cynomys ludovicianus*); GU, Gunnison's prairie dog (*Cynomys gunnisoni*); UT, Utah prairie dog (*Cynomys parvidens*); WT, white‐tailed prairie dog (*Cynomys leucurus*).

a Challenged with 10× higher dose.

All animals were housed in NWHC's BioSecurity Level‐3 animal isolation facility, and all animal protocols were approved by NWHC's animal care and use committee. Briefly, all prairie dogs were treated and housed similarly in every experiment (See Rocke et al., 2012 for details). Before transfer to NWHC, they were dusted with carbaryl and upon arrival examined for parasites and treated with an anthelminthic (Ivermectin) and Imidacloprid for additional flea control; the prairie dogs were gang‐housed on the floor with custom‐made stainless‐steel nest boxes and PVC pipes for refugia. Food (alfalfa‐based pellets and fresh vegetables) was provided once daily and water was provided ad libitum.

Challenge inocula of fully virulent *Y. pestis* strain CO92 were prepared as previously described (Osorio et al., 2003). Briefly, standard aliquots of *Y. pestis* of known MLD50 (confirmed to kill 50% of test mice), stored frozen in glycerol at −20°C, were thawed and diluted in 1× phosphate‐buffered saline (PBS) to achieve the desired dosage of challenge inoculum (in MLD50). Prairie dogs were restrained by hand and injected subcutaneously with 0.2 ml of the inoculum. For the larger dose (35,000 MLD50), the inoculum was divided and injected subcutaneously at six different injection sites, to simulate multiple flea bites. In every experiment, the challenge inoculum was also injected simultaneously in laboratory mice to confirm the intended dosage, and in every challenge, this dosage was achieved.

We used a Bayesian version of Prentice and Gloeckler's (1978) proportional hazards model for grouped (discrete) data (see Aune, Rhyan, Russell, Roffe, & Corso, 2012 for an example) to assess survival effects of species and geographic location where the prairie dogs were captured. In brief, the likelihood of the survival function can be written as $S\left(s|r\right)=\exp\left(-\sum_{i=r}^{s}\exp\left(\gamma +\beta_{1:k}*X_{k}\right)\right)$; where *S* is the rate of survival to time point *s* given survival to time point *r*, *γ* is the intercept value, *X* is a matrix of *k* covariates, and *β* is a vector of the *k* estimated coefficients. The unit cumulative hazard (the hazard from *r* to *s*) is $\Lambda =\exp\left(\gamma +\beta_{1:k}*X_{k}\right)$, and we estimated the coefficients *γ* and *β* using rjags (Plummer, 2013) in R (R Core Team, 2019). Priors for *γ* and *β* were chosen to be noninformative; they were normally distributed with a mean of zero and an inverse variance of 0.001.

In sum, for the first analyses, we compared the survival rates of prairie dog populations challenged with *Y. pestis*, including four Gunnison's prairie dog populations, two from Arizona and two from Colorado; seven black‐tailed prairie dog populations, five from South Dakota, one each from Colorado and Texas; one Utah prairie dog population from Utah; and one white‐tailed prairie dog population from Colorado. For the second analysis, we compared survival of white‐tailed prairie dogs from the same Colorado population challenged at two different dosages.

## 3 RESULTS

**Figure 2:**
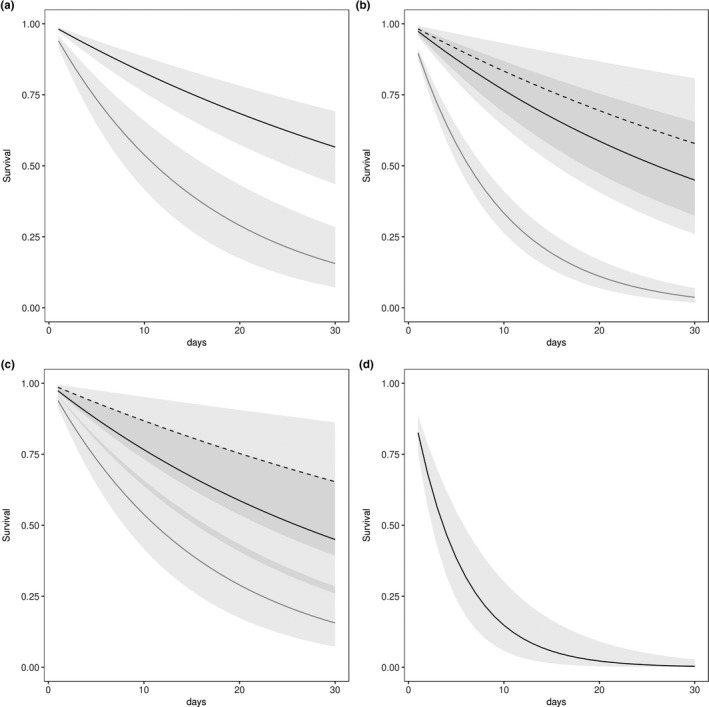
Estimated survival rates of prairie dogs challenged with *Yersinia pestis* in a laboratory setting (a) Gunnison's prairie dogs (*Cynomys gunnisoni*) from Arizona (solid black line) and Colorado (solid gray line), (b) black‐tailed prairie dogs (*Cynomys ludovicianus*) from South Dakota (solid gray line), Colorado (solid black line), and Texas (dashed black line), (c) black‐tailed (*C. ludovicianus*; solid black line), Gunnison's (*C. gunnisoni*; solid gray line) and white‐tailed prairie dogs (*Cynomys leucurus*; dashed black line) from Colorado, and (d) Utah prairie dogs (*Cynomys parvidens*). Light gray shading represents 95% confidence intervals

Overall, white‐tailed prairie dogs from Colorado had the largest percentage of survivors (67%) upon plague challenge at 3,500 MLD50 and the greatest number of mean days until death, 22.1 (2.26 *SE*; Table 2, Figure 2). No Utah prairie dogs from our single population survived plague challenge, and the average day of death was 5.7 (0.33 *SE*). Results of a Cox‐proportional hazard model indicate that Utah prairie dogs were less likely to survive challenge than all other populations of prairie dogs (Table 3, Figure 2). Estimated survival to day 30 for this population was 0.00 (95% Credible Interval [C.I.] 0.00–0.03). For black‐tailed prairie dogs, South Dakota populations were most susceptible to plague versus Colorado or Texas populations. Individuals from Colorado and Texas were 0.24 (95% C.I. 0.12–0.43) times and 0.17 (95% C. I. 0.06–0.35) times respectively less likely to die in a 1‐day interval than individuals from South Dakota populations. Estimated survival to day 30 for South Dakota black‐tailed prairie dogs from five populations was 0.04 (95% C.I. 0.02–0.07), for the population in Colorado, 0.44 (95% C.I. 0.25–0.65), and for the Texas population, 0.58 (95% C.I. 0.32–0.81). Arizona populations of Gunnison's prairie dogs were less susceptible than Colorado populations, and in Colorado, Gunnison's prairie dogs were more susceptible than black‐tailed prairie dogs. Estimated survival until day 30 was 0.16 (95% C.I. 0.07–0.28) for Gunnison's prairie dog populations in Colorado and 0.56 (95% C.I. 0.43–0.69) for populations in Arizona. White‐tailed prairie dogs from Colorado (from a single population) were equally susceptible to plague at equivalent challenge doses when compared to black‐tailed prairie dogs; however, sample size was small for this species and credible intervals were large (Table 3; Figure 2).

**Table 2:** Number of animals that survived or died from challenge with *Yersinia pestis* at a challenge dose of 3,500 MLD50 (except where noted) and mean day of death of animals that died

Location	Species	Survived	Died	% Survival	Mean day of death (*SE*)
Colorado	BT	12	12	50.0	8.9 (1.41)
South Dakota	BT	9	95	8.6	6.9 (0.27)
Texas	BT	9	6	60.0	10.3 (1.14)
Arizona	GU	36	24	60.0	10.4 (0.73)
Colorado	GU	11	29	27.5	5.8 (0.35)
Utah	UT	0	21	0.0	5.7 (0.33)
Colorado	WT	10	5	67.0	22.1 (2.26)
Colorado^a^	WT	0	10	0.0	6.4 (0.33)

Note

Values in parenthesis are standard errors.

Abbreviations: BT, black‐tailed prairie dog (*Cynomys ludovicianus*); GU, Gunnison's prairie dog (*Cynomys gunnisoni*); UT, Utah prairie dog (*Cynomys parvidens*); WT, white‐tailed prairie dog (*Cynomys leucurus*).

a Challenged with 10× higher dose.

**Table 3:** Parameter estimates and hazard estimates from Cox‐Proportional hazards analyses of survival data from prairie dogs challenged with *Yersinia pestis* at challenge doses of 3,500 MLD50. a) AZ, Arizona; BT, black‐tailed prairie dog (*Cynomys ludovicianus*); CO, Colorado; GU, Gunnison's prairie dog (*Cynomys gunnisoni*); UT, Utah prairie dog (*Cynomys parvidens*); TX, Texas; WT, white‐tailed prairie dog (*Cynomys leucurus*). b) Comparison of white‐tailed prairie dogs challenged at 3,500 and 35,000 MLD50

	Parameter estimates			Hazards		
	LCL 2.5%	Median 50%	UCL 97.5%	LCL 2.5%	Median 50%	UCL 97.5%
a)						
Intercept	−2.42	−2.21	−2.01			
Colorado BT versus South Dakota BT	−2.08	−1.41	−0.85	0.12	0.24	0.43
Arizona GU versus South Dakota BT	−2.22	−1.76	−1.32	0.11	0.17	0.27
Texas BT versus South Dakota BT	−2.76	−1.8	−1.04	0.06	0.17	0.35
Utah versus South Dakota BT	0.05	0.55	1.01	1.05	1.73	2.75
Colorado GU versus Colorado BT	0.19	0.84	1.57	1.21	2.32	4.81
Colorado WT versus Colorado BT	−0.42	0.34	1.14	0.66	1.4	3.13
b)						
Intercept	−5.32	−4.26	−3.47			
Standard versus high dose	1.40	2.45	3.65	4.06	11.62	38.57

**Figure 3:**
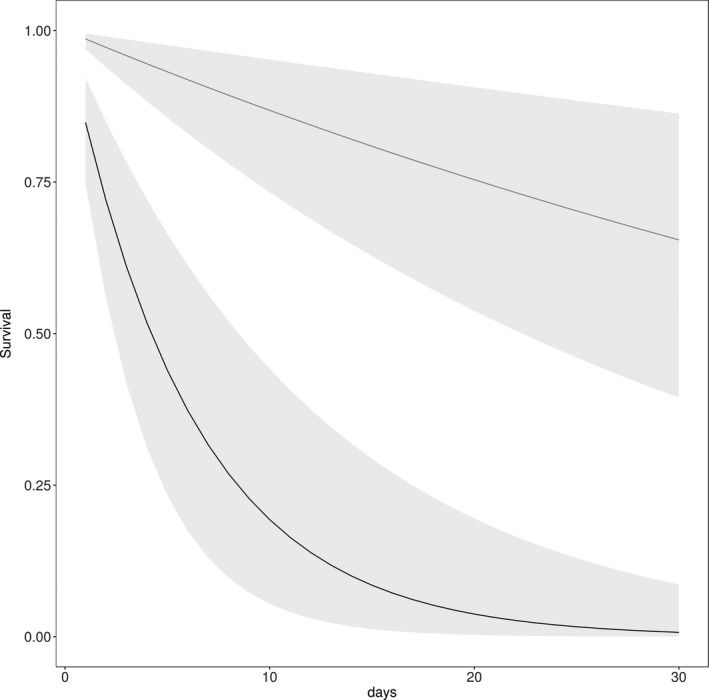
Estimated survival rates of white‐tailed prairie dogs challenged with *Yersinia pestis* at a challenge dose of 3,500 MLD50 (gray line) and 35,000 MLD50 (black line) in a laboratory setting. Light gray shading represents 95% confidence intervals

**Figure 4:**
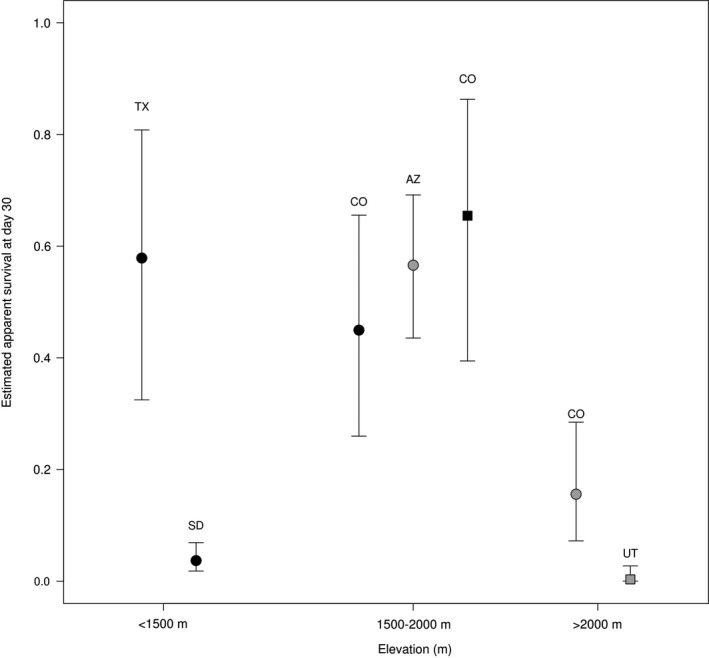
Estimated survival at day 30 for black‐tailed prairie dog (black circles) populations from Texas (TX), South Dakota (SD), Colorado (CO); Gunnison's prairie dog (gray circles) populations from Arizona (AZ), and Colorado; white‐tailed prairie dogs (black squares) from Colorado; and Utah (UT) prairie dogs (gray squares). Error bars represent 95% confidence intervals

White‐tailed prairie dogs challenged with a single injection of 3,500 MLD50 of *Y. pestis* were 11.62 x more likely to survive and survived longer (average 22.1 days; 2.26 *SE*) compared with animals that received multiple injections with a 10× higher dosage (average 6.4 days; 0.33 *SE*; Figure 3). Survival rates were highest for mid‐elevation populations that had experienced a long history of plague outbreaks, including black‐tailed and white‐tailed prairie dogs in Colorado, black‐tailed in Texas, and Gunnison's prairie dogs in Arizona (Figure 4). Survival rates were lowest for black‐tailed prairie dogs in South Dakota, without previous exposure to plague, and high‐elevation Gunnison's and Utah prairie dog populations (Figure 4.)

## 4 DISCUSSION

In laboratory challenge trials using a single strain of *Y. pestis*, we found differences in relative plague susceptibility between prairie dog species and also among populations within a species. Numerous factors might explain this differential susceptibility. Prairie dogs may be more adapted to a local strain of plague, suggesting different results might be obtained with a different challenge isolate. However, while some local diversification of *Y. pestis* isolates may occur (Lowell et al., 2015), all *Y. pestis* isolates in the Americas were clonally derived from a single isolate introduced in the early 1900s (Anisimov, Lindler, & Pier, 2004), and significant differences in virulence among isolates have not been previously described. Thus, we consider it unlikely that prairie dogs would display highly variable responses to different plague strains (i.e., be susceptible to some strains and not others).

Our results indicate that for at least one species, black‐tailed prairie dogs, differences in historical exposure to plague may influence plague susceptibility. Populations of black‐tailed prairie dogs in South Dakota, where plague was not detected until 2004, were more susceptible to plague than populations in Texas and Colorado, where plague first occurred in the 1940s and 1950s. However, despite a long history of plague exposure (Figure 1), Utah prairie dogs and Gunnison's prairie dogs from high‐elevation populations in Colorado were also highly susceptible to plague challenge, indicating that historical exposure does not explain resistance patterns in all localities.

For the endangered Utah prairie dog, which has experienced severe population declines due to overexploitation, habitat destruction, and disease (Brown, Peacock, & Ritchie, 2016), poor connectivity has resulted in small isolated sets of subpopulations. For Gunnison's prairie dogs, there is some evidence of isolation by elevation (Sackett et al., 2014). Though one population in Colorado occurred at a slightly higher elevation than the other, and possibly represents a different subspecies (*C. g. gunnison* compared to *C. g. zuniensis*; Sackett et al., 2014), both populations occurred at >2,000 m, and in previous analyses, no difference in plague susceptibility was detected between the two populations (Rocke et al., 2015). However, animals from these Colorado populations were more susceptible to plague than the Arizona populations which occurred at elevations <2,000 m. Possibly, the Colorado populations are more genetically isolated due to elevation or lack of connectivity than the populations in Arizona. This genetic isolation or population fragmentation can lead to lowered gene flow as observed in other systems (Jousimo et al., 2014), which may have inhibited the development of widespread resistance to plague. Our study is based on relatively few populations, and additional replication using colonies from these and other populations is necessary to determine how broadly these findings can be applied to populations across the range of the prairie dogs in North America.

Whatever the proximate cause for differential susceptibility (historical exposure or poor connectivity), it is likely that changes in host genetics are the mechanism by which these differences occur (Rocke et al., 2012). Several genetic loci in resistant laboratory mice are under investigation for their role in plague resistance (Chevallier et al., 2013; Demeure et al., 2012), and a possible association with MHC class II alleles in Gunnison's prairie dogs has been proposed (Cobble et al., 2016). If a genetic mechanism of resistance exists in prairie dogs, it is probably complex and polygenic. In wild populations, acquired humoral immunity has been suggested as the mechanism of survival for black‐tailed prairie dogs in Wyoming (Pauli et al., 2006). However, in the Rocke et al. (2008), Rocke et al. (2010), Rocke et al. (2012) studies, no antibody activity was recorded prior to *Y. pestis* challenge, and similarly, Busch et al. (2013) determined that mechanisms other than humoral immunity were probably responsible for differential plague susceptibility in Gunnison's prairie dogs in Arizona. A similar outcome was noted in rock squirrels (Quan et al., 1985).

Currently, questions remain regarding plague dynamics in prairie and shrub‐steppe ecosystems. Numerous hypotheses have been posed to explain where and how plague persists on the landscape between epidemics. Unlike other potential maintenance hosts, such as voles and grasshopper mice, the development of genetic resistance to plague in prairie dogs is considered unlikely due to the high mortality rates suffered by prairie dogs during plague outbreaks. Our data suggest that some level of plague resistance may occur in some prairie dog populations, providing a mechanism for plague to be maintained at low levels within a colony for a period of time or possibly to spread the plague bacterium (or infectious fleas) among closely neighboring colonies, as animals in varying stages of infection disperse from outbreak locations due to social upheaval (Hoogland, 2013). However, this mechanism is unlikely to result in completely resistant populations. In contrast, the recurring range‐wide plague die‐offs in all species of prairie dogs, even in populations that appear to have relatively lower susceptibility (e.g., Colorado and Texas), indicate this resistance is insufficient or can eventually be overwhelmed by local plague transmission dynamics.

At the standard challenge dose, numerous prairie dogs from some populations survived, especially white‐tailed prairie dogs. However, at a much higher challenge dose, white‐tailed prairie dogs from the same population succumbed to plague infection, illustrating that susceptibility to plague is dose‐dependent. In nature, flea abundance likely plays a role as increased flea abundance equates to potential for repeated bites and, thus, higher dosages of plague transmitted to prairie dogs. In addition, prairie dogs that survive an initial exposure to plague may succumb upon a subsequent exposure. The 10 white‐tailed prairie dogs that survived the standard plague challenge were challenged a second time 2 months later at a dosage of 12,000 MLD50, and six of the animals succumbed to infection. This population of white‐tailed prairie dogs suffered a plague epidemic during and after the time these individuals were captured, demonstrating their susceptibility to plague when naturally vectored by fleas. Therefore, our test subjects may have been exposed to plague prior to capture, although prechallenge antibody titers to plague were negative. These results may also be influenced by body condition or physiology of captive animals, with the animals in better condition being more likely to withstand plague challenge. Pauli et al. (2006) noted that prairie dogs surviving plague were in better body condition and hypothesized that survivors were either in better condition preplague or that reduced populations of prairie dogs postplague decreased competition for forage and led to increased body condition for survivors. In sum, genetic resistance is only one possible mechanism for survival of prairie dogs during plague epidemics.

Other factors that influence plague transmission are flea abundance and species composition, which are also related to environmental and seasonal (Tripp, Gage, Montenieri, & Antolin, 2009) conditions such as temperature and precipitation (Russell, Abbott, Tripp, & Rocke, 2018). If flea abundance on hosts is high, prairie dogs are likely to experience more flea bites and, therefore, may be more likely to contract plague. Our standard challenge dose is roughly equivalent to dosages delivered by several infectious flea bites (Burroughs, 1947; Eisen et al., 2008); however, our laboratory challenge studies did not account for multiple sources of infection or multiple and repeated flea bites, except for multiple plague injections in one group of white‐tailed prairie dogs. Our results, therefore, likely under‐represent mortality rates in wild prairie dog populations subject to outbreak conditions. Some flea species are more efficient at transmitting plague. For example, Wilder et al. (2008) found that *Oropsylla tuberculata cynomuris* was three times more efficient at transmitting plague than *Oropsylla hirsuta*. These flea species peak in abundance at different times seasonally (Tripp et al., 2009), and the composition and diversity of fleas varies geographically as well (Russell et al., 2018). Thus, even if certain populations of prairie dogs are apparently less susceptible to plague, they likely still experience high mortality rates from plague depending on seasonal variation in flea abundance and the flea species present.

### 4.1 Management implications

Prairie dogs form the basis of the endangered black‐footed ferret (*Mustela nigripes*) diet, and efforts to reintroduce black‐footed ferrets have been hampered by the decimation of prairie dog populations by plague. Prairie dog populations regularly exposed to plague may develop some level of resistance to the disease over time, and less susceptible prairie dogs may play a role in spreading plague or maintaining the bacterium between outbreaks. One potential outcome of lowered susceptibility is increased incubation time (Atkins, Townsend, Medlock, & Galvani, 2013; White, Reynolds, Waldron, Schneider, & O'Rourke, 2012) and thus increased time between exposure and infection. This is reflected in the mean day‐to‐death we observed in different species and populations of prairie dogs, ranging from 5.7 to 22.1 days at the same dosage of plague. Longer incubation times likely slow the time course of an outbreak, allowing it to smolder for months or even longer. Recent studies have demonstrated that plague can persist in individual populations at low levels prior to widespread mortalities (Salked et al., 2016; Tripp, Rocke, Runge, Abbott, & Miller, 2017). Thus, prairie dog populations that are not experiencing mass mortality events from plague may still be exposed or infected, and, managers should be aware that plague activity will likely go undetected in these situations. Cryptic sustained disease outbreaks have been observed in other disease systems as well (Salked et al., 2016) and suggest that sustained management of disease is necessary in the absence of observed outbreak conditions. Despite the apparent lowered susceptibility in some populations, recurring plague outbreaks in prairie dogs will continue to hamper conservation efforts for both prairie dogs and black‐footed ferrets.

## CONFLICT OF INTEREST

None declared.

## AUTHOR CONTRIBUTIONS

Dr. Tonie Rocke conceived and designed the overall study, Mr. Daniel Tripp contributed to the Colorado portion of the study. Dr. Robin Russell conducted the statistical analyses and wrote the manuscript. All three authors contributed substantially to revisions of the paper.

## Data Availability

These data are available at https://doi.org/10.5066/P9AZREUM (Rocke & Russell, 2019).
